# Prisoners’ access to HIV services in southern Malawi: a cross-sectional mixed methods study

**DOI:** 10.1186/s12889-021-10870-1

**Published:** 2021-04-28

**Authors:** Austrida Gondwe, Alemayehu Amberbir, Emmanuel Singogo, Joshua Berman, Victor Singano, Joe Theu, Steven Gaven, Victor Mwapasa, Mina C. Hosseinipour, Magren Paul, Lawrence Chiwaula, Joep J. van Oosterhout

**Affiliations:** 1 Dignitas International, P. O Box 1071, C/O Box 333, Zomba, Malawi; 2 University of North Carolina at Chapel Hill School of Medicine, Chapel Hill, NC USA; 3 University of Global Health Equity, Kigali, Rwanda; 4 College of Medicine, P/Bag 360, Chichiri, Blantyre, Malawi; 5 University of North Carolina-Malawi Project, Tidziwe Centre, P/Bag A-104, Lilongwe, Malawi; 6 Chichiri Prison, P/Bag 30117, Blantyre 3, Blantyre, Malawi; 7 Malawi Prisons Services, P.O Box 28, Zomba, Malawi; 8 David Geffen School of Medicine, University of California Los Angeles, Los Angeles, USA

**Keywords:** Prisoners, Malawi, HIV services, Female inmates, Risk behavior

## Abstract

**Background:**

The prevalence of Human Immunodeficiency Virus (HIV) among prisoners remains high in many countries, especially in Africa, despite a global decrease in HIV incidence. Programs to reach incarcerated populations with HIV services have been implemented in Malawi, but the success of these initiatives is uncertain. We explored which challenges prisoners face in receiving essential HIV services and whether HIV risk behavior is prevalent in prisons.

**Methods:**

We conducted a mixed-methods (qualitative and quantitative), cross-sectional study in 2018 in six prisons in Southern Malawi, two large central prisons with on-site, non-governmental organization (NGO) supported clinics and 4 smaller rural prisons. Four hundred twelve prisoners were randomly selected and completed a structured questionnaire. We conducted in-depth interviews with 39 prisoners living with HIV, which we recorded, transcribed and translated. We used descriptive statistics and logistic regression to analyze quantitative data and content analysis for qualitative data.

**Results:**

The majority of prisoners (93.2%) were male, 61.4% were married and 63.1% were incarcerated for 1–5 years. Comprehensive services were reported to be available in the two large, urban prisons. Female prisoners reported having less access to general medical services than males. HIV risk behavior was reported infrequently and was associated with incarceration in urban prisons (adjusted odds ratio [aOR] 18.43; 95% confidence interval [95%-CI] 7.59–44.74; *p* = < 0.001) and not being married (aOR 17.71; 95%-CI 6.95–45.13; *p* = < 0.001). In-depth interviews revealed that prisoners living with HIV experienced delays in referrals for more severe illnesses. Prisoners emphasized the detrimental impact of poor living conditions on their personal health and their ability to adhere to antiretroviral therapy (ART).

**Conclusions:**

Malawian prisoners reported adequate knowledge about HIV services albeit with gaps in specific areas. Prisoners from smaller, rural prisons had suboptimal access to comprehensive HIV services and female prisoners reported having less access to health care than males. Prisoners have great concern about their poor living conditions affecting general health and adherence to ART. These findings provide guidance for improvement of HIV services and general health care in Malawian institutionalized populations such as prisoners.

## Background

Globally, the prevalence of Human Immunodeficiency Syndrome (HIV) and other infectious diseases is much higher among prisoners than in the general population [1]. Although data is limited, East and Southern African regions have some of the highest reported HIV prevalence ratios among prisoners in the world [2]. In 2012, HIV prevalence in prison populations was 41% in South Africa, 28% in Cote d I’voire, 27% in Zambia, and in Zimbabwe it was suggested that more than half of prisoners were HIV infected [3]. Additionally, a systematic review and meta-analysis of global and cross-country prevalence of HIV among prisoners showed that HIV prevalence was highest in Africa [4]. Factors believed to be contributing to high HIV prevalence in prisons are overcrowding, limited access to health care, intravenous drug use and unsafe injecting practices, sexual violence, unprotected sex and tattooing [5].

Prison health care in Africa is under-resourced and much more funding is needed to ensure adequate HIV testing services and antiretroviral therapy (ART) [6]. Other challenges in sub-Saharan African prisons include limited human resources, fragmented referral systems that prevent continuity of care when detainees are discharged from prison or transferred to another prison, and overcrowding, contributed to by high rates of pre-trial detention [7]. A study conducted in Zambia and Uganda reported that while HIV testing had been extended in prisons in recent years, access to testing remained limited and treatment was inadequate [8]. Additionally, lack of knowledge amongst prisoners about risks of contracting and transmitting HIV, absence of protective measures, and proper medical care all increase prisoners’ risk of HIV infection [9].

Imprisoned people are frequently re-incarcerated after release, cycling between prison and the general community [10]. As potential carriers of transmissible diseases they pose risks to themselves, their immediate families and the wider community, with detrimental effects on public health [7, 11, 12]. HIV services such as counseling, treatment, care and support should therefore be part of a comprehensive health care program for all prisoners, in order to make it equivalent to comprehensive services that are available in the community [13]. The World Health Organization (WHO) states that in some settings recommended prison HIV prevention and treatment services are unavailable, despite the fact that HIV prevalence in prison may be 15 times higher than in the general adult population [13].

Limited data is available about HIV prevalence in Malawi’s prisons, with the most recent studies conducted more than 10 years ago [14] and no information exists about access to ART. According to a UNAIDS report, Malawi has made impressive progress to curb the HIV epidemic in the general population [2]. However, progress in making HIV testing and treatment services available in Malawian prisons has not been described recently. Therefore, we aimed to investigate prisoners’ self-reported access to HIV services in southern Malawi and the challenges they face in utilizing HIV prevention and care.

## Methods

### Study design and setting

We conducted a mixed-methods (quantitative and qualitative), cross-sectional study in six prisons in Southern Malawi. Malawi has 23 prisons, which include 4 central prisons and 19 district prisons, with total prison population of approximately 14,060 [15]. In addition, Malawi has two reformatory schools (Mpemba and Chilwa) for juveniles under the age of 15. HIV prevention and care policies for prisons in Malawi include HIV and sex education, Prevention of Mother to Child Transmission (PMTCT); voluntarily male medical circumcision (VMMC), and ART and HIV counseling and testing (HTC). Condom and lubricants distribution is excluded [16]. TB screening is generally implemented along with HIV interventions [16]. Male and female prisoners are strictly separated to different sections of the prison but have access to the same prison clinic. The current study took place in 6 prisons in southern Malawi, which contain more than 50% of the prison population in Malawi [17]. Two of the six are central prisons that often house more than 1200 prisoners despite having a capacity of 800. Dignitas International and Medicines San Frontiers were supporting the Ministry of Health with providing health services in the two central urban prisons and in two rural prisons. The study focused on the southern region because it was convenient for the study completion.

### Study sampling procedure

We recruited 424 study participants to establish the availability of HIV services based on the assumptions that HIV services are available among 50% of the study population [18], a 5% margin of error, a confidence level of 95%, and factored in a 10% non-response rate.

We obtained a list of prisoners from each prison and used a systematic random sampling technique to select prisoners for the quantitative part of the study. For the qualitative part, we used available lists of prisoners on ART that we received from warders and selected prisoners for in-depth interviews randomly from those lists. The inclusion criteria were age greater than 18 years and provision of informed consent. Based on information from prison officers, we excluded prisoners who were incarcerated in segregation housing (for instance due to being elderly or violent or due to specific prison criteria for isolation). We also excluded prisoners whose participation posed important logistical challenges, for example those who were considered dangerous to interviewers. Finally, prisoners who were too sick to participate in the study according to the warder’s opinion were excluded.

### Data collection

The study was approved by the University of Malawi College of Medicine Research Ethics Committee (P.02/17/211). All participants who took part in the study signed or thumb-printed an informed consent form. The interviews were conducted in the local language *Chichewa*. All data collected through the questionnaire and in-depth interviews were anonymized. Two experienced data collectors (one male, one female) were trained to administer a structured questionnaire using the Open Data Kit (ODK) electronic system for data collection. The questionnaire was administered in private circumstances and captured demographic characteristics of prisoners, availability of and access to HIV services within prisons and on risk behaviors that increase the transmission of HIV, such as unprotected sexual contact and unsafe use of needles and spikes. The questionnaire and the interview guide were first piloted and adjustments were made based on the interviewers’ feedback.

We requested to speak to prisoners living with HIV in the qualitative part of the study, to explore access to HIV services and associated challenges. The prison warders keep a list of prisoners who are HIV positive and we selected 39 prisoners living with HIV and on ART randomly to participate in in-depth interviews. Some already knew their positive HIV status before being imprisoned, and some were accessing ART outside prison facilities, with family members delivering their ART medication. The first author conducted all the in-depth interviews (duration 40–60 min each) using an interview guide. Interviews were conducted in a room that was outside hearing distance from the prison guards but with security maintained by having prison staff watching the interview process from a distance.

### Statistical analysis

We analyzed quantitative data using STATA software, version 14. Descriptive analysis (using proportions and frequency tables) was used to assess the range of HIV services available to prisoners. In-depth interviews were audio recorded, transcribed, translated and analyzed, using a content analysis approach. For example, the printed transcripts were reviewed and initial codes were created and combined into themes that were presented cohesively. Logistic regression was used to investigate associations between prisoner characteristics and risk behaviors in prisons, defined as having accepted or provided sex in exchange for money/goods/other favors and/or used tools such as spikes, razor blades and needles unsafely. The results of the quantitative study are presented first followed by presentation of qualitative studies. In some cases, we have presented respondents’ quotes verbatim when two or more respondents were of the same reported opinion and when the quote was of particular relevance.

## Results

### Quantitative study findings

**Fig. 1:**
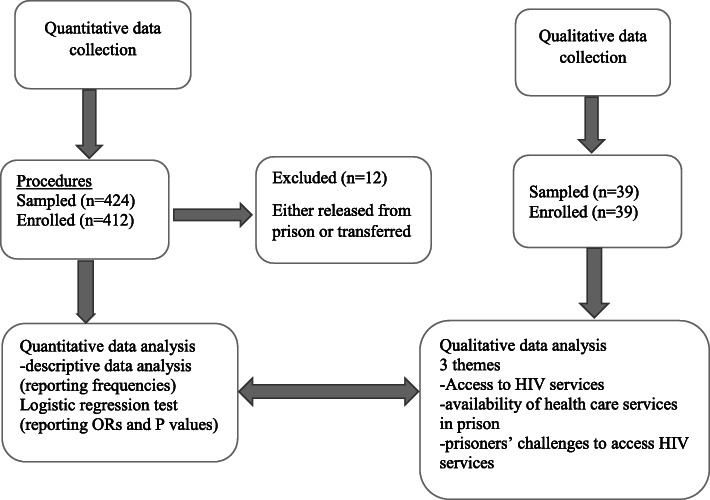
Flow chart for participants’ recruitment

Four hundred twelve prisoners were enrolled (Fig. 1) in the quantitative study during January and February 2018 (12 had been released from prison or transferred to another prison at the time of the study). The majority were male (93.2%) and younger than 35 years (87%). About 9% were on remand (i.e. not yet sentenced) and 63.1% were sentenced to serve for a period of 1 to 5 years. Detailed demographic characteristics of participants in the quantitative study are presented in Table 1.

**Table 1:** Participants demographic characteristics

Prisoners characteristics	Total n (%)	Central Urban Prisons n (%)^**a**^	Small Rural Prisons n (%)^**b**^
	*N* = 412	*N* = 353	*N* = 59
***Age category (years)***			
18–29	164 (39.8)	133 (37.7)	31 (52.5)
30–44	197 (47.8)	174 (49.3)	23 (39.0)
45+	51 (12.4)	46 (13.0)	5 (8.5)
***Sex***			
Male	384 (93.2)	327 (92.6)	57 (96.6)
Female	28 (6.8)	26 (7.4)	2 (3.3)
***Prison status***			
Remand	36 (8.7)	32 (9.1)	4 (6.8)
Sentenced/convicted	376 (91.3)	321 (90.9)	55 (93.2)
***Marital status (current)***			
Married	253 (61.4)	209 (59.2)	44 (74.6)
Single	73 (17.7)	66 (18.7)	7 (11.9)
Divorced/Separated/Widowed	86 (20.9)	78 (22.1)	8 (13.6)
***Source of income before prison***			
Employed	106 (25.7)	92 (26.1)	14 (23.7)
Unemployed	30 (7.3)	28 (7.9)	2 (3.4)
Business	276 (67.0)	233 (66.0)	43 (72.9)
***Period of incarceration (years)***			
< 1	76 (18.4)	58 (16.4)	18 (30.5)
1–5	260 (63.1)	222 (62.9)	38 (64.4)
> 5	76 (18.4)	73 (20.7)	3 (5.1)

^a^Urban prisons are defined as those located in the major cities

^b^Rural prisons are defined as those located in districts including satellite prisons of the urban prisons

There was limited variation in knowledge about HIV services between prisoners of urban and rural prisons (Table 2). Prevalence of reported HIV risk behaviors such as paying and receiving money for sex was generally low but higher in urban compared to rural prisoners (4.4% vs. 0%; *p* = 0.09). Sharing of tools such as razor blades was less common in urban prisons than in rural prisons (52.7% vs. 74.6%; *p* = 0.001).

**Table 2:** Prisoners’ knowledge of HIV Services and experience of HIV risk behaviour^a^

	Urban Prison *n* = 353	Rural Prison *n* = 59	*P* value
*N* = 412	Yes n (%)	Yes n (%)	
**Access to HIV information**			
HIV	297 (84.1)	50 (84.7)	0.91
Condoms	186 (52.7)	44 (74.6)	< 0.001
Lubricants	31(8.8)	15 (25.4)	< 0.001
Tuberculosis	297 (84.1)	51 (86.4)	0.65
Antiretroviral Therapy	294 (83.3)	47 (79.7)	0.49
PMTCT	110 (31.2)	21 (35.6)	0.50
Clean needles and syringes	236 (66.9)	47 (79.7)	0.05
Voluntary Counselling and Testing	305 (86.4)	51 (86.4)	0.99
HIV positive living^c^	324 (91.8)	54 (91.5)	0.95
Male circumcision	255 (72.2)	47 (79.7)	0.23
**Information on HIV risk behaviors**			
Provided sex to others in prison	24 (7.9)	1 (3.0)	0.31
Paid/accepted money/goods for sex	16 (4.4)	0 (0.0)	0.09
Shared needles/spikes and syringes^b^	51 (16.8)	7 (21.2)	< 0.001

^a^HIV risk behaviors include actions that would influence the spread of HIV in prisons

^b^Needles and spikes: Sharp objects used for tattooing and piercing of ears

^c^HIV positive living: include any information about how those who are HIV positive can take care of their lives

Table 3 shows results of prisoners’ access to HIV services including comprehensive screening and information services. In urban prisons, there was a significant difference on HIV voluntary counselling and testing between the urban and rural prisons (88.4% vs. 98.3%; *p* = 0.02). Prisoners in urban prisons reported having more access to HIV services, including HIV testing (82.4% vs. 57.6%; *p* = < 0.001) and TB screening inside the prison (81.0% vs. 66.1%; *p* = 0.01). Prisoners reported that post-exposure prophylaxis (PEP) was not available in any of the prisons.

**Table 3:** Access to HIV/AIDS services

	Urban Prison *n* = 353	Rural Prison *n* = 59	*P* value
*N* = 412	Yes (n%)	Yes (n%)	
***Are the following HIV services available to prisoners in your prison?***			
HIV voluntary counseling and testing	312 (88.4	58 (98.3)	0.02
Screening for Tuberculosis	341 (96.6)	57 (96.6)	0.10
Treatment of Tuberculosis	340 (96.3)	56 (94.9)	0.60
Prevention of mother to child transmission of HIV (PMTCT)	39 (11.1)	3 (5.1)	0.16
Antiretroviral Therapy (ART) for HIV	340 (96.3)	57 (96.6)	0.91
Male circumcision	175 (49.6)	33 (55.9)	0.37
Supplementary feeding for HIV or TB patients	90 (25.5)	15 (25.4)	0.99
Condoms	44 (12.5)	3 (5.1)	0.1
HIV post-exposure prophylaxis	0 (0)	0 (0)	N/A
***Use of HIV services in prison***			
Tested for HIV inside the prison	291 (82.4)	34 (57.6)	< 0.001
Screened for TB inside the prison	286 (81.0)	39 (66.1)	0.01

Results of multivariable logistic regression analysis of factors associated with reported HIV risk behaviors showed that this risk was higher with prison location in urban than in rural prisons (adjusted OR [aOR] 17.71; 95% - CI 6.95–45.13; *p* = < 0.001). Married status was associated with a lower risk (aOR 0.37; 95% - CI 0.14–0.97; *p* = 0.043). However, age group, being on remand vs. convicted status, and duration of incarceration were not significantly associated with HIV risk behavior (Table 4).

**Table 4:** Factors associated with presence of HIV risk behavior in prisons^b^

Variables	Risk behaviour Yes (%)	Crude OR (95% CI)	*P*-value	Adjusted OR (95% CI)^a^	*P*-value
**Sex**					
Male	259 (78.5)	**21.89 (2.59–184.77)**	**0.005**	**26.60 (2.81–251.50)**	**0.004**
Female	1(14.3)	Reference		Reference	
**Age group in years**					
18–29	97 (74.1)	Reference	–	Reference	–
30–44	135 (79.9)	1.39 (0.81–2.39)	0.232	1.74 (0.87–3.50)	0.118
45 and above	28 (75.7)	1.09 (0.47–2.54)	0.841	1.18 (0.42–3.29)	0.748
**Prisoners status**					
Remand	17 (77.3)	Reference	–	Reference	–
Sentenced/convicted	243 (77.1)	0.99 (0.35–2.78)	0.989	1.00 (0.27–3.50)	0.999
**Marital status**					
Single	55 (85.9)	Reference	–	Reference	**–**
Married	156 (74.3)	**0.47 (0.22–1.02)**	**0.056**	**0.37 (0.14–0.97)**	**0.043**
Separated/divorced/ widowed	49 (77.8)	0.57 (0.23–1.44)	0.236	0.42 (0.14–1.28)	0.128
**Duration of incarceration in years**					
< 1	33 (68.8)	Reference	–	Reference	–
1–5	171 (77.0)	1.52 (0.77–3.03)	0.228	0.81 (0.33–2.01)	0.651
> 5	56 (83.6)	2.31(0.95–5.63)	0.064	0.85 (0.28–2.58)	0.779
**Prison location**					
Urban	253 (83.2)	**18.43 (7.59–44.74)**	**< 0.001**	**17.71 (6.95–45.13)**	**< 0.001**
Rural	7 (21.2)	Reference		Reference	

^a^Model adjusted for sex, age, location of prison, prisoner status, marital status and period of incarceration

^b^Risk behavior is defined as having either accepted or provided sex in exchange for money/goods/other favors and/or unsafe use of tools such as spikes, razor blades and needles

### Qualitative results

Among the 412 prisoners, we enrolled 39 who were HIV positive for in-depth interviews. One female prisoner refused to be audiotaped for fear of her HIV status getting disclosed despite signing the confidentiality form. In the analysis, three sub-themes emerged; i) access to HIV services, ii) availability of health care services in prison, and iii) prisoners’ challenges to access HIV services. We discuss these sub-themes below.

### Availability of health care services in prison

In the two urban prisons, prisoners reported that they had a well-established on-site clinic that was providing various services including HTC, ART, TB screening, hepatitis B screening and care for other, general diseases. Prisoners reported being offered HTC at point of entry and regularly during incarceration, which helped them to know their HIV status. In the same urban prisons, prisoners said they trusted the quality of HTC due to the involvement of NGOs in the clinics. Additionally, they also expressed concerns about sustainability if these organizations would decide to stop supporting HIV services. One prisoner in an urban prison felt that HIV testing at the point of prison entry was compulsory and not optional as highlighted below;

“*It was by compulsory because they were doing it cell by cell. Whether you like it or not, you were being tested*” (Prisoner -006).

In two rural prisons, the prisoners complained that HTC services were only offered outside prison premises, including HIV testing. Prisoners in the urban prisons reported having ready access to ART and those found HIV positive were immediately assisted to start ART. Some prisoners in the urban prisons believed that the medical care in prisons was not different from what was provided outside the prison setting.

*I am also one of them who have been on ART for long time and have been in prison for so many years. For example, when I look back in 2007/2008 it was difficult to find ARVs but in 2016 things changed. We have seen things changing for the better and now we can find the ARVs without difficulties* (Prisoner, -037).

On the other hand, prisoners in one of the rural prisons complained that they are not provided with ART regularly. Lacking an on-site clinic, they also did not get sufficient chance to access ART in the nearby hospital.

### Access to HIV services

While adequate access to ART, TB screening and other services was reported to be available in the urban prisons, some prisoners reported delays in referring seriously ill prisoners to main hospitals. They believed that in some cases this had led to deaths which could have been prevented. Only prisoners from one particular urban prison reported getting timely help for any illnesses.

*Right here, they assist us very well when we are sick but the only problem is that sometimes it takes them long time to assist us. Even if you report to the officers that you are sick, it takes them time to refer you to the main hospital. Many die in the cells because the prison officers neglect us very much* (Prisoner, 003).

In urban prisons, female prisoners reported that they had delayed access to the clinic for any type of sickness. Female prisoners believed that their male counterparts had better access to medical services. For example, one female prisoner felt that the health workers at the prison clinic are negligent in handling sick females and believed that sometimes they receive treatment without adequate diagnosis:

*We females do not have direct access to the clinic unlike the males. They think we will start having relationships and I don’t know how but as women, we don’t have that chance of being treated at our own prison clinic and they tell us that is part of security. They do not look at the signs but just give us drugs. For us, it is challenging because even if we report on those who are sick it is not immediate that they will come and check on them. It takes them a day or two to come and check on the sick ones. Sometimes they just advise us to take water which I feel is not good. They are supposed to take us to hospital but they don’t want to do so despite that we have a clinic here inside the prison.* (Prisoner 015)

### Challenges to access HIV services

We asked prisoners if they had any challenges to access the HIV services within the prison setting. Prisoners mentioned that the most important challenges they faced were not related to access to HIV services but were problems relating to their living conditions. In all prisons, prisoners complained of poor sanitation. For example, female prisoners reported lack of sanitary pads and poor bathrooms. Some prisoners believed that being on ART whilst in prison was a big challenge because of the poor diet such as having one meal per day and sometimes having no meal at all. HIV positive prisoners believed that good diet was important especially for those on ART. In all prisons, male prisoners viewed that overcrowding was a challenge and some prisoners reported having stopped taking their ARVs due to lack of private space to keep their drugs. One prisoner who was also working as an HIV peer educator explained;

*Like me, I am a guardian for someone else but I am also taking the medication. That friend we are very close but he refuses to keep the medication on his own. He clearly says that I do not want to keep this medication so I keep it for him. When it is time to take the medication I take the medication and give it to him and I am the one who encourages him but I am also one of the people taking the medication. There are many people who are on medication but do not keep it for fear to be known.* (Prisoner 002)

Prisoners reported that risk behaviors (tattooing, sharing of razor blades, and sex inside prison) among prisoners could influence the spread HIV/AIDS. For instance, one urban prisoner reported that some male prisoners were trapped into having sexual relationships with fellow prisoners to obtain favors such as space to sleep and money for their needs. Participants further reported that there was high chance of contracting TB whilst in prison because of overcrowding. They mentioned that the very limited cell space led to those on treatment being mixed in the same cell with other prisoners. One female prisoner believed that lack of protective measures such as gloves would put them at risk to HIV as women in their cell were often fighting and could sometimes bleed.

## Discussion

In a large, mixed-methods study among prisoners in southern Malawi, prisoners generally reported having adequate information about the main aspects of HIV and HIV services, but also indicated having knowledge gaps on the availability of condoms, PMTCT and SRH issues. Prisoners in larger urban prisons with on-site, NGO-supported clinics reported having better access to some HIV services including HIV testing, VMMC, condoms and TB screening and treatment than prisoners in smaller urban and rural prisons. As opposed to unsafe use of razor blades and needles, sex in prison was uncommon and was more commonly reported by urban than by rural prisoners. In multivariable analysis, urban prison location was associated with higher overall HIV risk behavior. In in-depth interviews, prisoners living with HIV reported several challenges to accessing HIV/AIDS services, including delayed referral to hospitals for more severe illnesses, and particularly in rural prisons, lack of on-site availability of HIV services, such as VMMC, PEP and supplementary feeding during illness. Female prisoners mentioned that they had less access to health care and HIV services than male counterparts. All emphasized the importance of poor general living conditions such as inadequate sanitation, lack of food, insufficient diet and overcrowding, and said this also hampered taking ARVs adequately.

Our study shows that reported access to a wide range of HIV services depended on the presence of well-established, on-site clinics supported by NGOs, as found in the two large urban prisons. At such clinics, good-quality ART care outcomes can be achieved as demonstrated by a cross sectional study conducted in Zomba Central Prison (one of the current study’s prisons), showing good virological suppression rates under challenging circumstances [18]. Although the urban prisoners appreciated the NGO support of the on-site clinics, they also expressed concerns about their long-term sustainability in case external funding would cease. These findings underline the importance of expansion of comprehensive HIV services to smaller, rural prisons with emphasis on sustainability through increasing governmental investments. Regular outreach mobile clinics may be considered to achieve this, similar to existing community ART distribution services.

Our study found that female prisoners were having less access to general medical care and HIV services than men. Similar findings were reported in several sub-Saharan African countries [10]. A study conducted in Ghana found that female prisoners reported poor access to quality healthcare and that their perceptions were influenced by marital status, educational background, and occupation [19]. A study from Zambia found that female prisoners’ access to healthcare was limited due to lack of in-house clinics, weak responsiveness by prison officers to their requests for healthcare and a general favor to male prisoners for providing health services [20, 21]. A scoping review of literature reported a lack of support for female prisoners’ needs around menstruation in prisons in Namibia, Uganda, Mozambique, Malawi, Cameroon, Ethiopia, Zimbabwe, Zambia, Nigeria and South Africa [10]. Taken together, these findings indicate that more attention has to be given to the health care of African female prisoners, including for HIV and reproductive health services [1].

Various studies reported risk behaviors that may increase the spread of HIV in prisons [22]. Although we found no association between duration of incarceration with such risk behaviors, in in-depth interviews prisoners felt that poverty triggered some prisoners to practice sex in exchange of favors such as space to sleep and better food from those who have been imprisoned for long periods. Policies and regulations in Malawi are known to inhibit condom use in prisons because homosexuality is illegal and is believed not to take place in prison cells [16]. For these reasons, sexual activity may have been underreported by the participants. Regardless, these policies may strain efforts towards HIV control in Malawian prisons and instead a variety of improvements of prison conditions may be needed to prevent forced sexual encounters and transactional sex.

As in other reports from prisons in sub-Saharan Africa our study participants emphasized the impact of poor living conditions on their general health [7] and they reported that this also affects their ability to adhere to ART. Overcrowding in prison cells, poor sanitation, and the effect of insufficient diet on the immune system can all have detrimental effects on the spread of infections within prisons. Improvements of general conditions in prisons will benefit the health of prisoners and will also provide general public health benefit when transmission of chronic infections such as TB, HIV and hepatitis B from ex-prisoners into general populations is prevented.

The main strength of our study is that we combined quantitative methods with in-depth interviews allowing us to have a detailed understanding of HIV prisoners’ experiences in accessing HIV services. The study was large with very few exclusions and refusals. Participant prisoners were from different types of prisons, increasing the representativeness of the findings. Additionally, the confidentiality measures likely promoted accurate reporting of services. We used digital recording of the in-depth interviews, which enhanced the quality of the analysis of the views of prisoners about access to HIV services. We managed to avoid some challenges of interviewing prisoners, such as trying to change the focus of the study and diverting to what feels more important to them [22]. There were several limitations to our study. We did not enroll very sick and potentially violent prisoners, and we used available lists of HIV positive prisoners provided by warders to select participants for in-depth interviews, which may have biased our findings. Our study was not designed to establish prevalence of HIV risk behavior and its reporting by prisoners is sensitive to several potentially disturbing factors. While we made efforts to mitigate these as much as possible, our HIV risk findings should be regarded with caution. We did not do in-depth interviews with HIV negative prisoners as we placed emphasis on ART services in the qualitative part of the study. We only included prisons from the southern region of Malawi and this may limit the generalizability of our findings.

## Conclusion

Prisoners in Southern Malawi reported adequate general knowledge about HIV services with some gaps in specific areas. HIV risk behavior was noted by limited numbers of prisoners, mostly in urban prisons. Prisoners from two large urban prisons with on-site, NGO-supported health facilities had access to a wide range of HIV services, indicating substantial progress achieved over the last decade [18], but availability of HIV prevention and care was still suboptimal in smaller rural prisons. Female prisoners complained that they had less access than men to health care, including HIV services. Prisoners living with HIV reported that poor living conditions and overcrowding affects their general health and personal HIV management. These findings provide guidance for improvement of HIV services and general health care in Malawian prisons.

## Data Availability

All data generated or analysed during this study are included in this published article [and its supplementary information files].

## Abbreviations

AIDS: Acquired Immune Deficiency Syndrome
ART: Antiretroviral therapy
HIV: Human Immunodeficiency Syndrome
HTC: HIV Testing and Counseling
NGO: Non-Governmental Organization
TB: Tuberculosis
